# The effects of PIKfyve inhibitor YM201636 on claudins and malignancy potential of nonsmall cell cancer cells

**DOI:** 10.3906/biy-2010-32

**Published:** 2021-02-09

**Authors:** Eda DOĞAN, Zekeriya DÜZGÜN, Zafer YILDIRIM, Berrin ÖZDİL, Hüseyin AKTUĞ, Vildan BOZOK ÇETİNTAŞ

**Affiliations:** 1 Department of Medical Biology, Faculty of Medicine, Ege University, İzmir Turkey; 2 Department of Medical Biology, Faculty of Medicine, Giresun University, Giresun Turkey; 3 Department of Histology and Embryology, Faculty of Medicine, Ege University, İzmir Turkey

**Keywords:** Claudin, epidermal growth factor receptor, nonsmall cell lung cancer, PIKfyve, YM201636

## Abstract

PIKfyve is an evolutionarily conserved lipid and protein kinase enzyme that has pleiotropic cellular functions. The aim of the present study was to investigate the effects of phosphatidylinositol-3-phosphate 5-kinase (PIKfyve) inhibitor, YM201636, on nonsmall cell lung cancer (NSCLC) cells growth, tumorigenicity, and claudin (CLDN) expressions. Three NSCLC cell lines (Calu-1, H1299 and HCC827) were used to compare the effects of YM201636. Cytotoxic effects of YM201636 were analysed using XTT assay. Malignancy potential of cells assesses with wound healing and soft agar colony-forming assays. mRNA and protein expressions of claudins were analysed by qRT-PCR and immunofluorescence staining. Our results revealed that YM201636 inhibited the proliferation and malignancy potential of Calu-1, H1299, and HCC827 cells in a dose-dependent manner. After YM201636 treatment CLDN1, -3 and -5 expressions increased significantly in HCC827 cells. CLDN3 and -5 expressions also significantly increased in Calu1 cell line. YM201636 treatment significantly reduced the CLDN1 and increased the CLDN5 expression in H1299 cells. Immunofluorescence staining of CLDN1, -3 and -5 proteins showed a significant increase after YM201636 treatment. Besides, YM201636 induced EGFR mRNA expression in all NSCLC cell lines. Our results have shown that YM201636 inhibits tumorigenicity of NSCLC cells. Furthermore, estimated glomerular filtration rate (EGFR) pathway is important signalling involved in the regulation of claudins. Understanding the mechanisms of PIKfyve inhibitors may improve cancer treatment particularly for EGFR overactivated NSCLC.

## 1. Introduction

Lung cancer is one of the most common cancer type that is expected to be diagnosed in men and women in 2020 (Siegel, et al. 2020). Nonsmall cell lung cancer (NSCLC) accounts for 85% of all lung cancer cases. Delayed first diagnosis, chemotherapy resistance and aggressiveness of the tumour cells are still major obstacles in the lung cancer treatment, and novel therapeutic agents are urgently needed.

PIKfyve (phosphoinositide kinase, FYVE-type zinc finger containing) is an evolutionarily conserved lipid and protein kinase that has pleiotropic cellular functions (Shisheva, 2008). Phosphorylation of the phosphatidylinositol-3-phosphate (PI3P) by PIKfyve generates phosphatidylinositol 3,5-bisphosphate [PtdIns(3,5)P2] or phosphatidylinositol 5-phosphate (PtdIns5P) (Shisheva, 2001). These two phosphoinositide (PI) derivatives have been proposed to be involved in: intracellular membrane trafficking pathways (Ikonomov et al., 2006; Sbrissa et al., 2007), translocation of the glucose transporter GLUT4 after insulin stimulation (Sbrissa et al., 2004), regulation of synapse strength (McCartney et al., 2014), remodelling the actin cytoskeleton (Ikonomov et al., 2018), and epidermal growth factor receptor (EGFR) signalling (Kim et al., 2007). Besides the physiological roles, PIKfyve has been associated with oncogenesis and cancer cell migration (Oppelt et al., 2014; Oppelt et al., 2013). Therefore, inhibition of PIKfyve activity has been started to explore as an anticancer therapy. 

YM201636 compound was discovered as a selective small-molecule inhibitor of PIKfyve leading to the accumulation of a late endosomal compartment and blockade of retroviral exit (Jefferies et al., 2008). YM-201636 mediated PIKfyve inhibition significantly reduced the survival of primary mouse hippocampal neurons and promoted vacuolation of endolysosomal membranes followed by apoptosis-independent cell death (Martin et al., 2013). It was demonstrated that YM201636 blocks the continuous recycling of the tight junction proteins claudin-1 (CLDN1) and claudin-2 (CLDN2) (Dukes et al., 2012). Tight junctions (TJs) are intramembrane multiprotein complexes that provide apical intercellular connections between adjacent cells in both epithelial and endothelial monolayers (Shin et al., 2006). In particular, barrier functions of TJs are important for lung defence; so they are considered as a part of the innate immune system (Soini, 2011). 

Claudins are structural molecules of TJs and responsible for the regulation of the paracellular permeability of cells (Salvador et al., 2016). Alteration of claudin expressions in lung tumours have been reported by several studies; however, the effects of PIKfyve inhibitor YM201636 on NSCLC cells have not been investigated. Thus, we aimed to investigate the effects of YM201636 on NSCLC cells proliferation, malignancy potential, and expressions of CLDN1, -3 and -5, which are “pore-sealing” claudins, and increased expression of them leads to increased tightness of epithelial monolayer and transepithelial electrical resistance and decreases permeability (Krause et al., 2009).

## 2. Materials and methods

### 2.1. Cells and reagents

To compare the effects of YM201636, we used three NSCLC cell lines (Calu-1, H1299, and HCC827) with different genotypic backgrounds. Calu-1 and H1299 are carcinoma cell lines carrying homozygous TP53 deletion and lack of expression of p53 protein. HCC827 cell line possesses EGFR mutation (exon19del E746-A750), which leads to constitutively activated EGFR. 

All cell lines were obtained from the American Type Culture Collection (ATCC, Manassas, USA). Cell culture media and supplements were obtained from Biological Industries (Bio Ind. Kibbutz Beit Haemek, Israel). Calu-1 cells were cultured in DMEM (Dulbecco’s modified Eagle’s medium); HCC827 and H1299 cells were cultured in RPMI-1640 medium in a humidified 37 °C incubator with 5% CO_2_. Both medias were supplemented with 10% FBS (fetal bovine serum), 1% L-glutamine, and penicillin-streptomycin.

YM201636 was obtained from Cayman Chemical (#13576) and dissolved in DMSO (final concentration 10 mM). For mRNA expression analysis primer, probe and enzymes were purchased from Roche Applied Science (Mannheim, Germany). 

### 2.2. Cytotoxicity assay

Calu-1, H1299, and HCC827 cells were seeded into 96-well plates at a density of 2×10^4^ per well. The next day, cells were treated with increasing concentrations of YM201636 (1, 2.5, 5, 7.5, 10, 12.5, 15, and 20 µM). Control groups were treated with the DMSO according to the concentration in the YM201636. After 24, 48, and 72 h, 50 µL of XTT solution (Bio Ind. Kibbutz Beit Haemek, Israel) was added to each well, and the plate was incubated for 4 h. The absorbance of each well was measured spectrophotometrically at 450 nm with an ELISA reader (Thermo Fisher Scientific, Massachusetts, USA). All concentrations were triplicated and experiments repeated at least 3 times. The growth inhibition percentage was calculated as follows: 

Growth inhibition % = (OD_control_ - OD_YM201636_)/OD_control_ × 100

### 2.3. Quantitative reverse-transcription polymerase chain reaction (qRT-PCR)

mRNA expression patterns of CLDN-1, -3, -5 and EGFR were assessed by quantitative real-time PCR. qRT-PCR analysis was performed on the LightCycler™ 2.0 instrument (Roche Applied Science, u1de8). Relative quantification of each sample with glyceraldehyde-3-phosphate dehydrogenase (GAPDH) was achieved by using different standards included in each run.

### 2.4. Immunofluorescence staining

Calu-1, H1299, and HCC827 cells were cultured on 15 mm cover glasses (5x10^5^ cells/mL). After 24 h incubation, cells were fixed in 4% paraformaldehyde (Sigma, #P-6148) for 30 min and treated with 0.25% Triton X100 (Bio Basic Canada Inc. C34H62O11) for 15 min and blocked with 1% bovine serum albumin (BSA, Chem Cruz, #sc2323) in 1× phosphate-buffered saline (PBS). Primary antibodies, claudin-1 (Proteintech, #22952-1-AP), claudin-3 (Santa Cruz, #sc271631), and claudin-5 (Proteintech, #22023-1-AP) were diluted 1/100 and incubated at 4 oC overnight. Secondary antibody (Invitrogen Alexa Fluor 488, #A11034 anti-rabbit) was diluted 1/200 and incubated for 1 h. Samples were mounted with Fluoroshield Mounting Medium with DAPI (Abcam, #ab104139) and observed by the appropriate fluorescent filter by Olympus CellSens Entry.

### 2.5. Wound healing assay

Wound healing assay was used to measure the migration of NSCLC cells. For this purpose, Calu-1, H1299, and HCC827 cells were seeded in six-well plates at a density of 5x10^5^ cells in 2 mL medium per well. Twenty-four-hours later, the cell monolayer was scratched with a sterile 100 μL pipette tip, then washed twice with PBS to remove debris which was followed by the addition of fresh medium that contained IC_50_ concentrations of YM201636. After 24 and 48 h, the gap distance was photographed using an inverted light microscope.

### 2.6. Soft agar colony-forming assay

Anchorage-independent growth assay was performed using the Cytoselect 96-well Cell Transformation Assay Kit (Cell Biolabs, # CBA-130). Calu-1, H1299, and HCC827 cells (3 × 10^4^) with various concentrations of YM201636 were seeded onto 96-well plates. After 7 days, colonies were counted and photographed.

### 2.7. Statistical analysis

IC_50_ concentrations of YM201636 were calculated with the GraphPad Prism. Statistical analyses were performed using IBM SPSS Statistics version 21 (IBM Corp., Armonk, NY, USA). One‑way analysis of variance (ANOVA) followed by Bonferroni or Dunnett’s T3 test was used for post hoc multiple comparisons. A two‑tailed unpaired t‑test was used for the comparison of the mean values between two groups. P < 0.05 was considered as statistically significant.

## 3. Results

### 3.1. Cytotoxic effects of YM201636 on NSCLC cell lines

**Figure 1:**
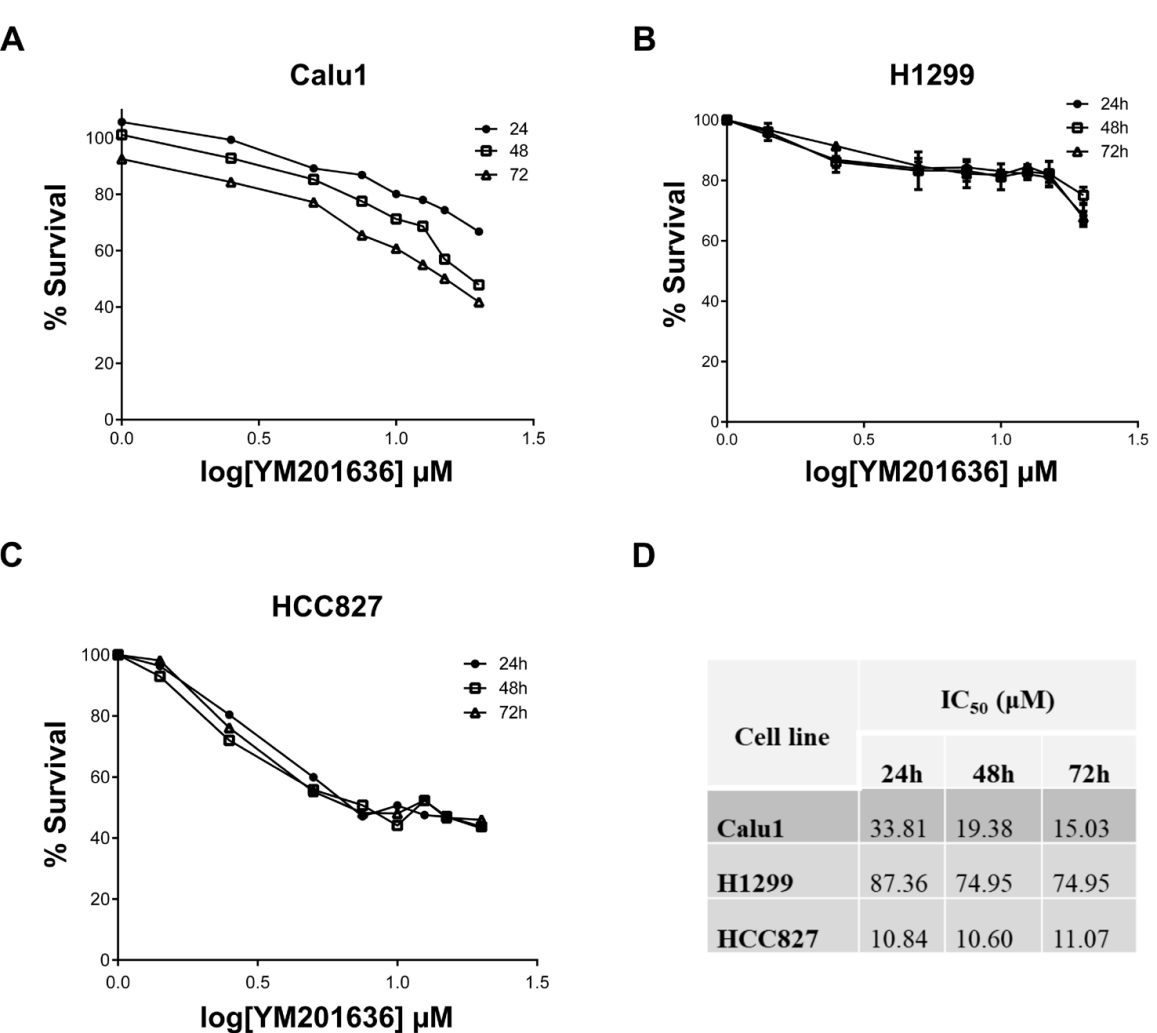
Effects of YM201636 on NSCLC cells proliferation. XTT assay was performed using A-) Calu-1, B-) H1299, and C-) HCC827 cell lines. D-) After 24, 48, and 72h exposure IC_50_ levels were calculated using GraphPad Prism 5.

To analyse the cytotoxic effects, NSCLC cells were treated with different concentrations of YM201636. After incubation for 24, 48, and 72 h, the viability was assessed using XTT cytotoxicity assay. Our results indicate that YM201636 decreased the viability of all cell lines (Figure 1). However, Calu1 and HCC827 cells were more sensitive to YM201636 with the 15.03 and 11.07 μM IC_50_ at the 72 h, respectively. Whereas H1299 cell line had 74.95 μM IC_50_ level for YM201636.

### 3.2. YM201636 induces Claudin-1, -3 and-5 expressions

**Figure 2:**
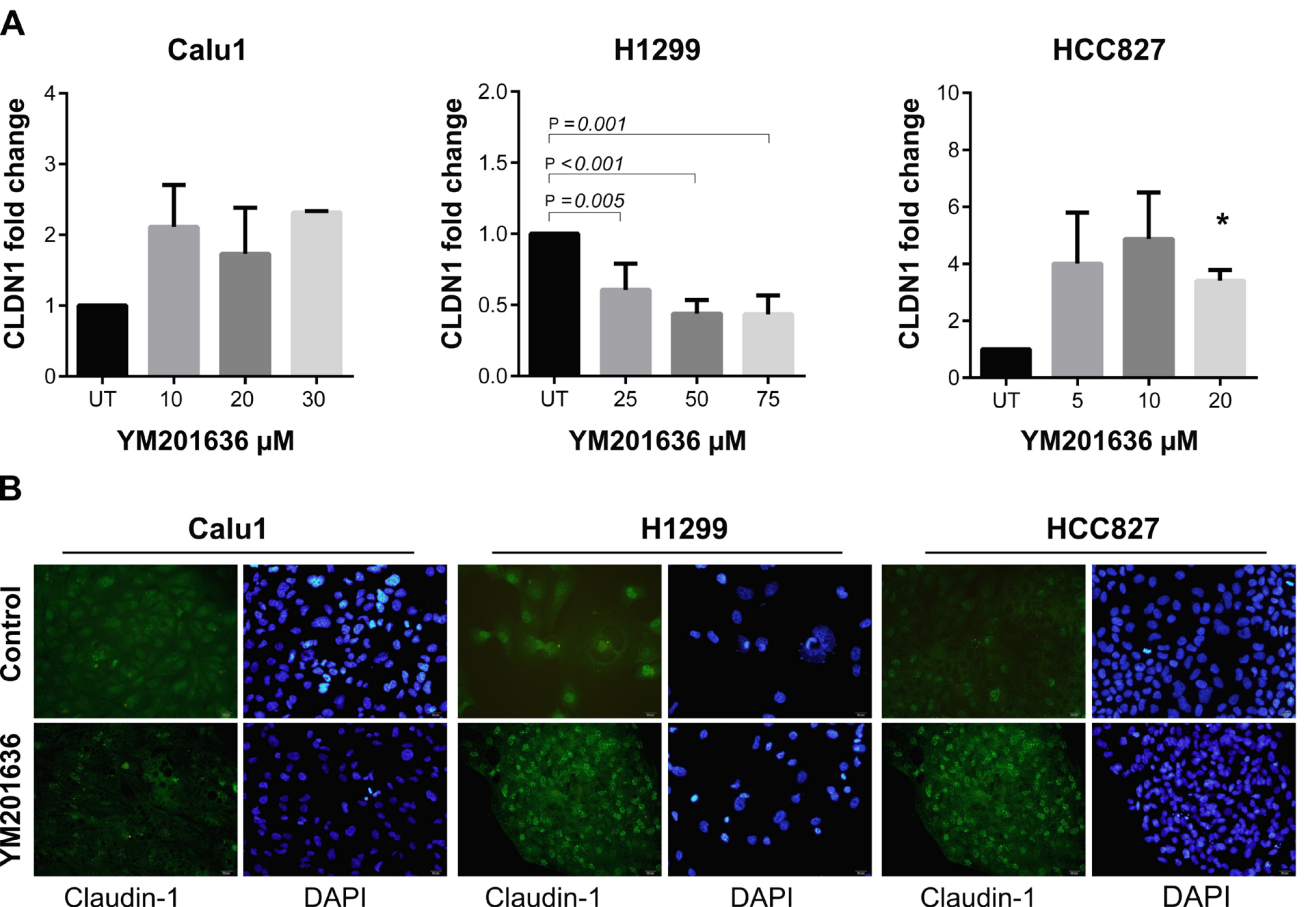
Effects of YM201636 on Claudin-1 expression. A-) mRNA analysis of CLDN1. Cells were treated with different concentrations of YM201636 (Calu-1: 10, 20, and 30 μM; H1299: 25, 50, and 75 μM; HCC827: 5, 10, and 20 μM) for 48 h. UT: Untreated cells. *P = 0.001, UT vs 20 μM. B-) Immunofluorescence staining of CLDN1 protein in NSCLC cells treated with YM201636 at IC_50_ doses. ×40 magnification.

**Figure 3:**
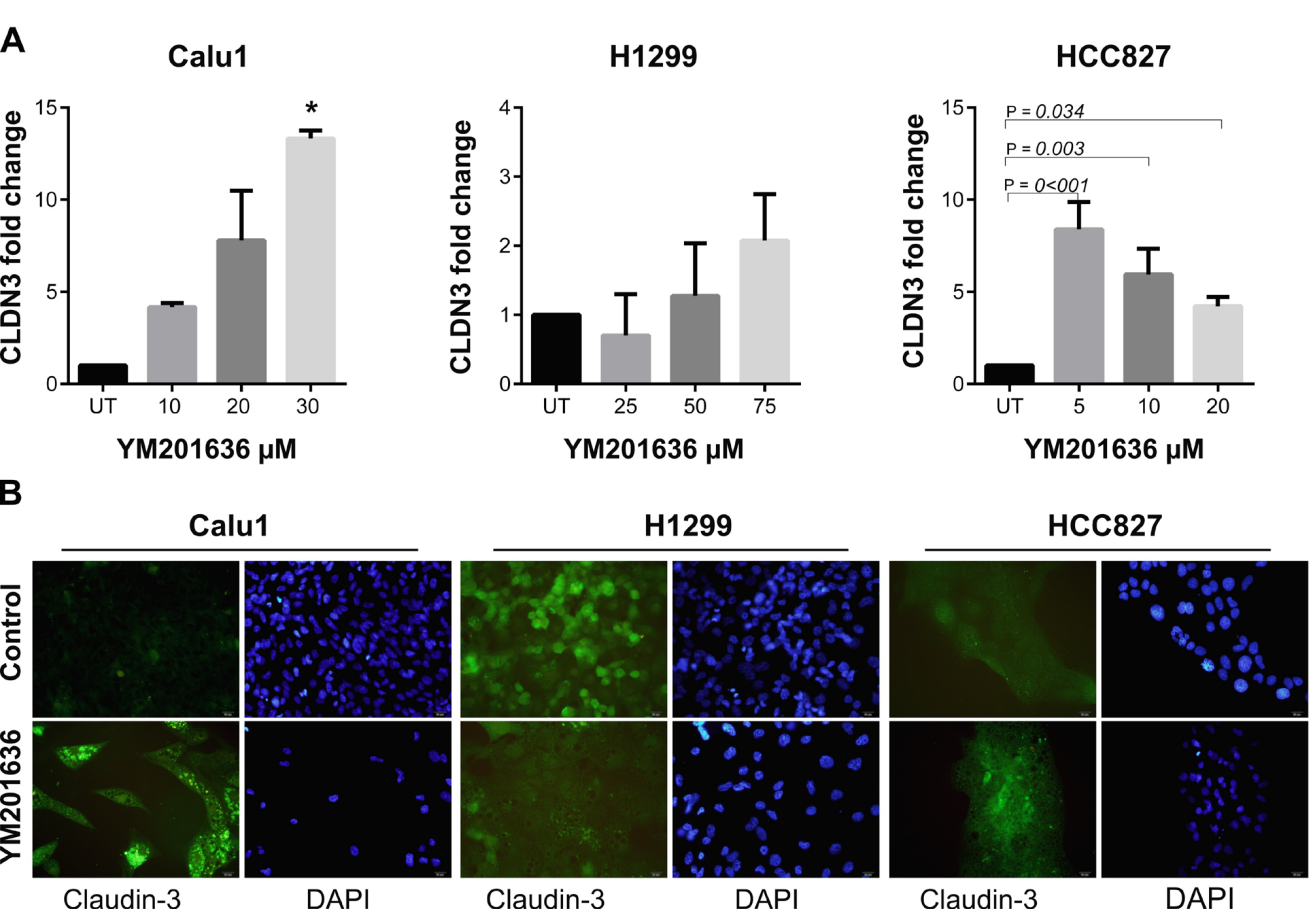
Effects of YM201636 on Claudin-3 expression. A-) mRNA expression and B-) immunofluorescence staining of CLDN3 in NSCLC cells after YM201636 treatment. UT: Untreated cells, *P = 0.007, UT vs. 30μM, ×40 magnification.

**Figure 4:**
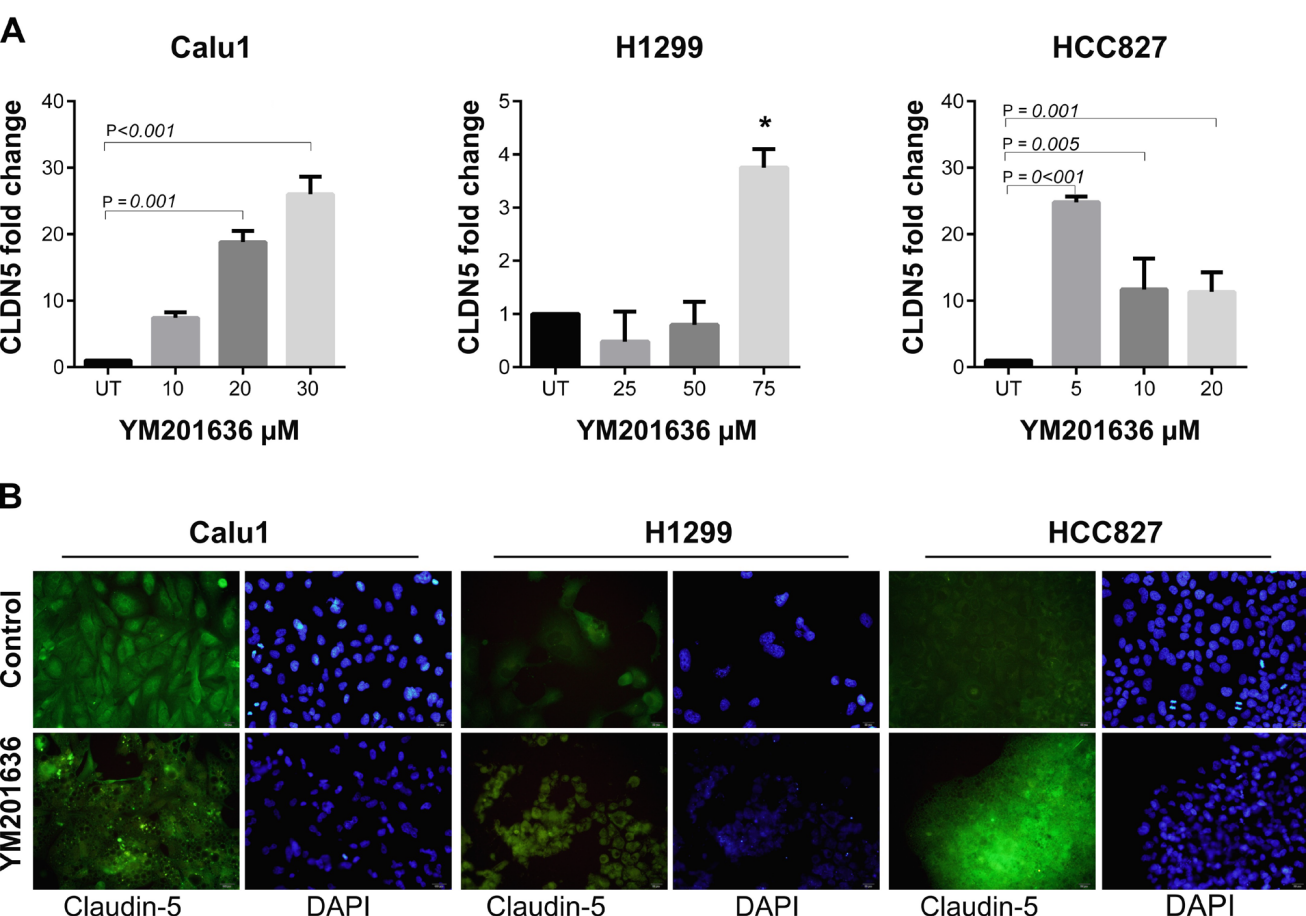
Effects of YM201636 on Claudin-5 expression. A-) mRNA expression and B-) immunofluorescence staining of CLDN5 in NSCLC cells after YM201636 treatment. UT: Untreated cells, *P = 0.015, ×40 magnification.

Previous studies have demonstrated that inhibition of PIKfyve via YM201636 treatment blocks the endocytic recycling of claudins (Dukes et al., 2012). We aimed to evaluate the effects of YM201636 on claudin-1, -3, and -5 expressions at the transcriptional level. Our qRT-PCR results showed that CLDN1 expression was significantly increased in HCC827 cells after 20 µM YM201636 treatment (Figure 2). However, CLDN1 expression tended to decrease with dose dependent manner in H1299 cells. On the other hand, CLDN3 and CLDN5 expressions were increased in all cell lines after YM201636 treatment (Figures 3 and 4). Except the CLDN3 expression in H1299, all increments were statistically significant for CLDN3 and -5. Furthermore, immunofluorescence staining of claudin-1, 3, and 5 proteins showed a significant increase after YM201636 treatment (Figure 2a, 3a and 4a). 

**Figure 5:**
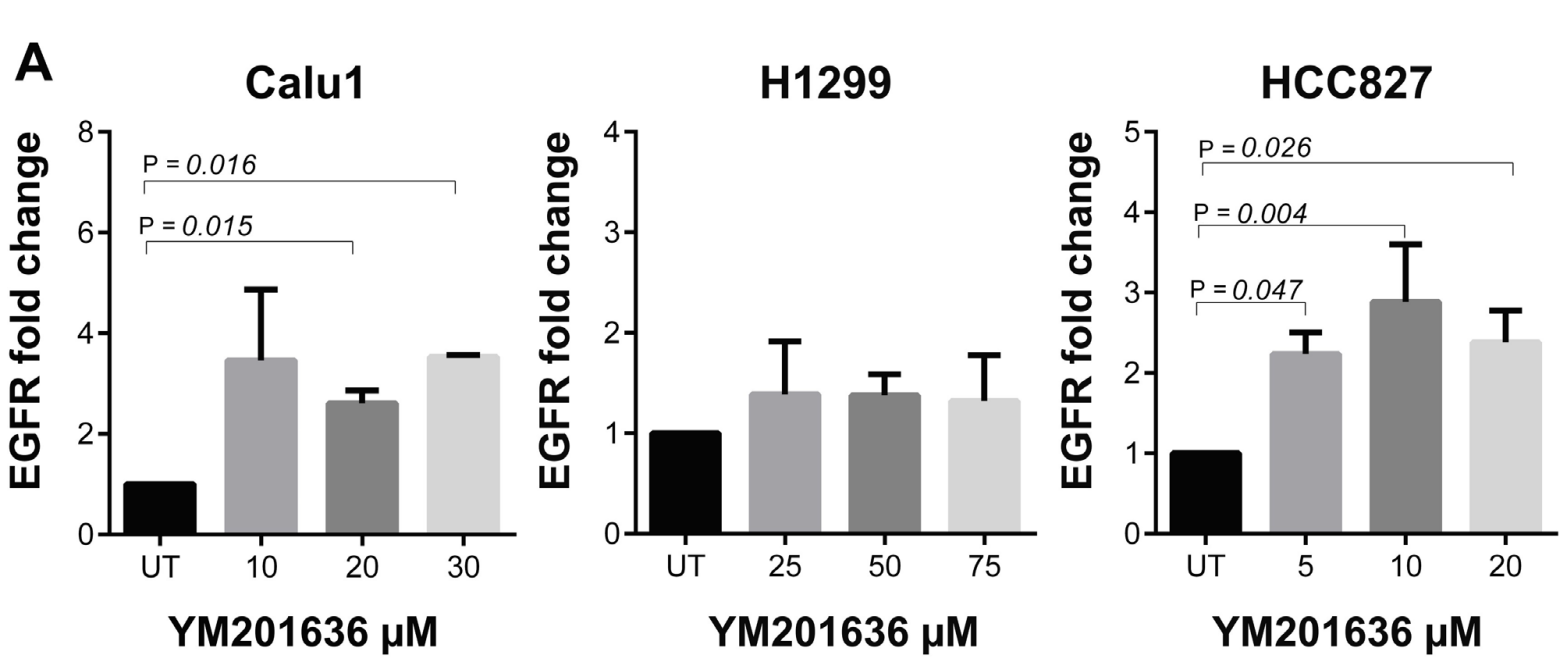
YM201636 induced EGFR mRNA expression in NSCLC cells. A-) Calu-1, B-) H1299, and C-) HCC827 cell line.

Hou et al. demonstrated that YM201636 suppressed the growth of liver cancer cells via the induction of autophagy upon EGFR overexpression (Hou et al., 2019). Therefore, we analysed the EGFR expression after YM201636 treatment. Our results showed that EGFR expression was increased in all cell lines after YM201636 treatment and increment was significant for Calu1 and HCC827 cells (Figure 5). 

### 3.3. Colony formation and cell migration after YM201636 treatment 

To study the effects of YM201636 on malignity potential of NSCLC cells, we analysed the anchorage-independent growth and cell migration.

**Figure 6:**
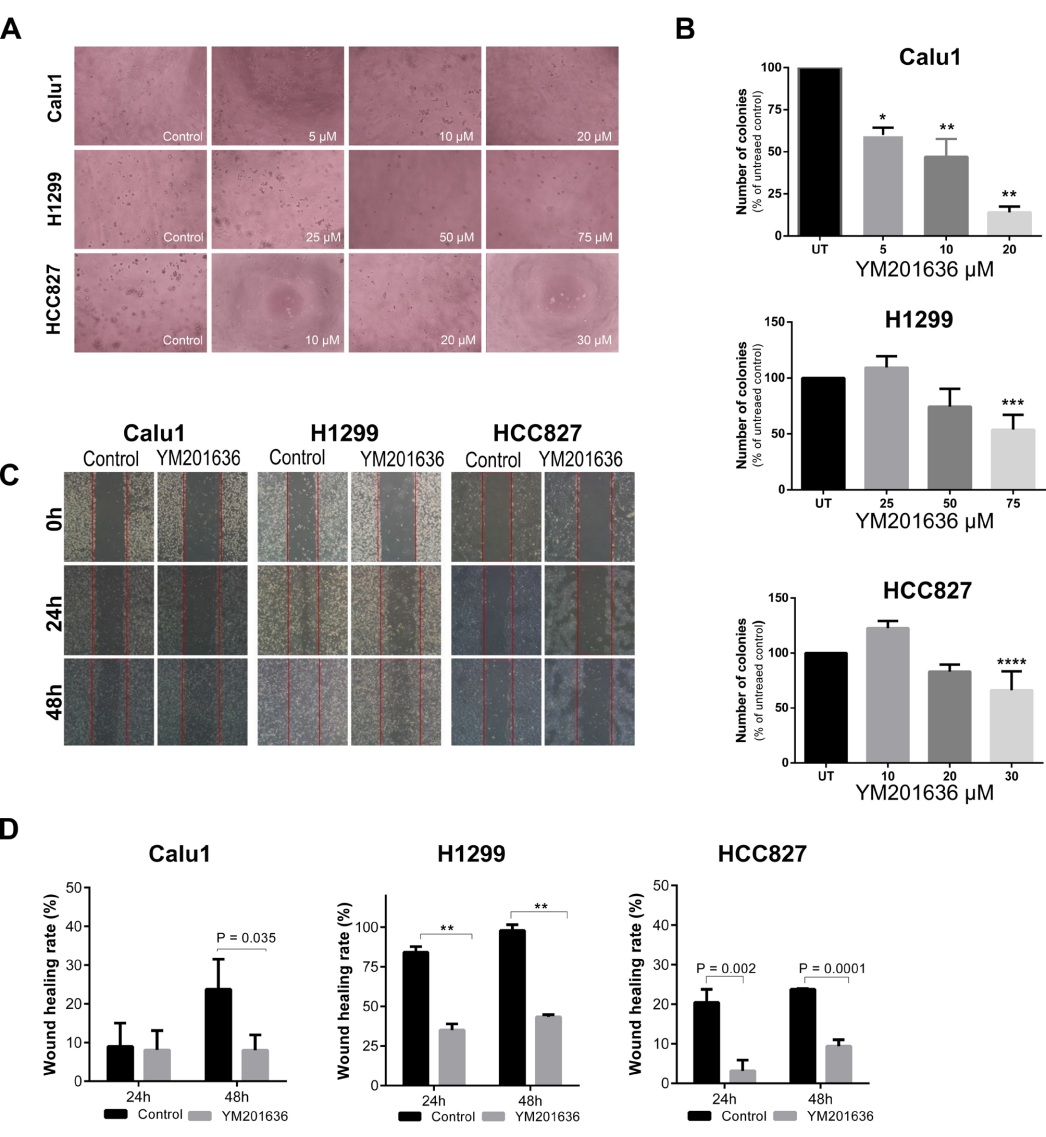
Effects of YM201636 on the tumorigenicity of NSCLC cells. A-) Colony-formation assay. Cells were treated with different concentrations of YM201636 (Calu-1: 10, 20, and 30 μM; H1299: 25, 50, and 75 μM; HCC827: 5, 10, and 20 μM). Colonies were counted and photographed on the 7th day. B-) Quantification of the number of colonies normalized to control cells. (*P = 0.0002, **P < 0.0001, ***P = 0.005, ****P = 0.006, Dunnett’s test) C-) Wound healing assay of NSCLC cells treated with YM201636 at the IC50 doses. Images were taken at 0, 24, and 48h. D-) Wound healing images were analysed using the Image J software to quantification of the areas. The graph shows the average values and the standard error of three experiments. **P < 0.0001.

For the anchorage-independent growth, cells were treated with 3 different concentrations of YM201636. As shown in the figure 6a - b, YM201636 treated Calu1 cells displayed significant decrease in the number of anchorage-dependent colonies at all doses (P < 0.0001). However, YM201636 treatment inhibited the colony formation only at the highest dose for H1299 (P = 0.005) and HCC827 (P = 0.006) cells. 

The capacity of cell migration was evaluated by wound healing assay as shown in Figures 6c and 6d. The monitored wound closure showed that YM201636 treatment significantly inhibited migration at Calu1 (P = 0.035), H1299 (P < 0.0001) and HCC827 (P = 0.0001) cells after 48h incubation. For the 24h, significant inhibition was detected at the H1299 (P < 0.0001) and HCC827 (P = 0.002) cells. 

These results demonstrated that exposure to the YM201636 significantly decreased the malignity potential of cells; therefore, PIKfyve kinase inhibition could be a target in NSCLC.

## 4. Discussion

In this study, we have used the PIKfyve inhibitor YM201636 to investigate the effects on proliferation, malignity potential, and claudin expressions in the NSCLC cell lines. Cytotoxicity assays show that YM201636 decreased the viability of all cell lines and Calu1 and HCC827 cells were more sensitive to YM201636. Different sensitivity to YM201636 may be due to genetic background of cells, and we speculated that EGFR overactivated cells were more sensitive to PIKfyve inhibition. 

It was demonstrated that cytotoxicity of PIKfyve inhibitors requires growth factor/Akt suppression and excessive cytoplasmic vacuolation (Ikonomov et al., 2018). PIKfyve inhibitors disrupted lysosomal function, autophagic flux, and the high basal necessity of autophagy in plasma cells and multiple myeloma cells (de Campos et al., 2020). Therefore, inhibition of PIKfyve was suggested as a novel target for multiple myeloma. In addition, Hou et al. reported that YM201636 possess an inhibitory effect on tumour cell growth via the induction of autophagy in the hepatoma cell lines and in vivo models without any adverse effects (Hou et al., 2019). Our results showed the cytotoxic effects of YM201636 on the NSCLC cell lines and supported previous studies. 

YM201636 inhibited the endocytic recycling of claudin-1 and -2, leading the accumulation of them intracellularly and delays epithelial barrier formation (Dukes et al., 2012). However, claudin-4 showed negligible endocytosis, and no detectable intracellular accumulation occurred following treatment with YM201636, suggesting that not all claudins show the same rate of endocytic trafficking. 

Claudins are structural proteins of TJs and localisation and expression of them regulate several cell functions (Martin and Jiang 2009). The distinct claudin expression profile was reported within the histologic subtypes of NSCLC (Moldvay et al., 2007). Lung adenocarcinoma patients with positive CLDN1 expression had poorer prognosis than negatives, and combination of CLDN1/Ras/EGFR was reported as a valuable independent prognostic factor (Sun et al., 2016). On the contrary, CLDN1 positivity predicted a better survival in adeno and squamous cell carcinoma (Merikallio et al., 2011; Moldvay et al., 2017). CLDN3 expression was significantly increased in lung adenocarcinoma tissues and it was associated with cancer progression and poor survival (Zhang et al., 2017). CLDN3 was reported as a positive regulator of cancer stemness in nonsquamous NSCLC and its depletion decreased the formation rates of spheres and tumours and increased cisplatin sensitivity (Ma et al., 2019). In brief, lung tumours with different histological types vary in their claudin expression showing up or downregulation of different claudins. It may not be sufficient to evaluate only claudin’s expression to predict cancer progression.

Since previous studies showed that YM201636 inhibited the endocytic recycling of claudins (Dukes et al., 2012), we evaluated the claudin expressions after YM201636 exposure. Our results showed that CLDN1 expression was significantly increased in HCC827 cells; CLDN3 and CLDN5 were significantly increased in Calu1 and HCC827 cells after YM201636 treatment. These results show that YM201636 affects the expression of claudins differently for each cell lines. YM201636 treatment induced all claudins (CLDN-1, -2, and -3) in EGFR mutant HCC827 cell line, which was the most sensitive to YM201636. Interestingly, YM201636 treatment reduced CLDN1, increased CLDN5 but did not change CLDN3 and EGFR expressions in H1299 cells, which is the less sensitive cell line to YM201636. 

EGFR might be the key point for the regulation of claudin transcription. It was reported that EGF and its downstream signalling pathway MEK/ERK or PI3K/AKT mediated the upregulation of CLDN3 expression and maintain the basic level of claudins (Zhang et al., 2017). Furthermore, YM201636-induced autophagy is dependent upon EGFR overexpression (Hou et al., 2019). Our study is consistent with previous results in terms of YM201636 induced EGFR. Promoter regions of CLDN1 and CLDN3 contain Sp-1 transcription factor binding site, which is crucial for their activation (Khan and Asif 2015). It was reported that EGFR signalling enhances IRES-mediated translation of Sp1 during tumorigenesis, and Sp1 accumulates in cancer cells (Hung et al., 2014). This explains the increase of claudins particularly in EGFR overactivated HCC827 cell line. Likewise, YM201636-induced autophagy was associated with the presence of EGFR (Hou et al., 2019). In this case, YM201636 induced EGFR protein was in an inactive form and it was demonstrated that inactive EGFR complexes could initiate autophagy (Hou et al., 2019; Tan et al., 2015).

EGFR activation significantly inhibited CLDN2 expression while increased CLDN1, -3, and -4 expression in MDCK cells (Singh and Harris, 2004). In addition, EGF-induced alterations in claudin expressions significantly amplified transepithelial resistance, which is a functional biomarker for TJs. EGFR inhibition is a common targeted therapy for EGFR over expressing tumours. It was reported that EGFR inhibitor gefitinib induces barrier function in human epidermal keratinocytes via the modulation of the expression of claudins (Fang et al., 2019). EGFR-PKC-CLDN1 pathway suggested as a tumorigenic pathway in colon cancer cells (Kim et al., 2020). These results clearly show that EGFR can both up- and downregulate claudin expressions and effect TJs functions. However, further studies are needed to find out the therapeutic potential of EGFR-tight junction connection.

There are some limitations of our study; 26 claudins are known to be expressed by humans, but we could not analyse all of them (Soini, 2011). Also, we cannot analyse autophagy markers and downstream proteins of the EGFR pathway. We are planning to investigate detailed mechanisms with further studies.

Overall, we have indicated that inhibition of PIKfyve using YM201636 resulted in an inhibitory effect on NSCLC cells growth and tumorigenicity. Furthermore, EGFR pathway is an important signalling cascade involved in the regulation of claudins. In conclusion, understanding the mechanisms of PIKfyve inhibitors may provide beneficial pharmacological combinations for improved efficacy particularly in the treatment of EGFR overactivated NSCLC. 
